# Does Any Analogy Exist Between Insanity and Demoniacal Possession?

**Published:** 1855-07-01

**Authors:** Jos. Souter

**Affiliations:** Chaplain to the Essex County Lunatic Asylum.


### DOES ANY ANALOGY EXIST BETWEEN INSANITY AND DEMONIACAL POSSESSION?

BY THE REV. JOS. SOUTER,

*Chaplain to the Essex County Lunatic Asylum.*

We have often heard the opinion, indeed it is asserted with a good deal of confidence, by some modern believers in the mystery of table-turning, that all insane persons are under the possession of devils. There are others, on the contrary, who are no less positive in their conviction that the influence of Satan in the matter, in ancient as well as modern times, is altogether a myth and an exploded folly. These latter hold that the demoniacs were merely insane. Both these opinions, though so utterly at variance with each other, start from the same point of some supposed and seeming analogy between insanity and possession. Whatever may be thought of the reasonableness of either of the above *conclusions* that have been drawn from it, it is certainly worth while to consider what grounds there are for supposing that such analogy exists. Believing this subject to be one of considerable interest, we propose, without further preface or apology, to enter upon its consideration, in a spirit alike removed, we trust, from an unreasoning bigotry on the one hand, and from the irreverence of a too bold rationalism on the other.

At the very threshold of this inquiry, it is of great importance that we should endeavour to ascertain whether the Evangelists make any special distinction between lunatics and demoniacs. We believe it to be universally acknowledged that no *example* is given of the cure of insanity, as called by that or any analogous name, to distinguish it from possession in any of the Gospels. But the twenty-fourth verse of the fourth chapter of St. Matthew is triumphantly appealed to by many writers, as proving that such cures were performed, and that no analogy between the two maladies can possibly be shown to exist. And certainly this passage forms a very plausible, if not unanswerable argument in favour of that opinion. We will quote the Evangelist's words:—"There were brought to Christ, both those which were possessed with devils, and those which were lunatic, καὶ δαιμονιζομένους καὶ σεληνιαζομένους, and he healed them." Valpy's note on this passage is most positive. He says,—"The persons possessed with devils are here *expressly distinguished* from lunatics, and could not be exactly the same." Many writers, both before and since the appearance of Dr. Valpy's Greek Testament, have maintained the same opinion; indeed, it has almost come to be regarded as treason or heresy to entertain a doubt about it. Dr. Adam Clarke expresses the utmost contempt and pity for those who are irrational enough, or infidel enough, to believe that the demoniacs were merely insane.

It is clear that the words already quoted from St. Matthew do, in a certain sense, distinguish the demoniacs and lunatics from each other; and if this distinction could be shown to be of as positive a nature as it has been supposed to be, we readily admit that the question in the mind of every devout believer in revelation would be set at rest. But we hope to be able to prove that the distinction made by the Evangelist is not of this nature, and does not militate at all against the opinion that the two diseases are analogous. Now it is worthy of remark, that the word translated "lunatic," occurs in only two places in the New Testament. Both these passages are in the same gospel; and it may, therefore, be assumed that the same meaning is attached to the word in each case. But in the latter of the two passages, the Evangelist uses the word lunatic and demoniac interchangeably. To the same man he applies both terms; or at least, he says of him, when our Lord had healed him, *ἐξῆλθεν ἀπ' αὐτοῦ τὸ δαιμόνιον*, "the demon departed from him." St. Luke also, and St. Mark, in whose gospels this miracle is recorded, both speak of him as a demoniac. It is therefore clear that in *one* sense the two classes were not distinct at all. Both were possessed.

We will now proceed to inquire, therefore, what meaning this word *σεληνιαζόμενος*, or lunatic, has; and since the passage quoted first from St. Matthew distinguished it from demoniac, in what respect it is that the two differ. We refer our readers to the seventeenth chapter of St. Matthew, as showing in what sense that Evangelist understood and used the word. A "lunatic" is brought to our Lord; the symptoms of his disease, and the circumstances attending his cure, are briefly recounted. What are these symptoms? The child's father describes them: he says this lunatic son was "deaf and dumb; he fell often into the fire and into the water." If we turn to the parallel place in St. Mark, we find him still further described as being "torn"—or rather as the word *ῥήσσει* signifies, and is translated in the margin—"dashed"—thrown upon the ground; and "he foameth, and gnasheth with his teeth, and pineth away." St. Luke adds yet another word to the description of this case:—"When the spirit taketh him, he crieth out, and it throws him into convulsions," *σπαράσσει αὐτόν*. It is scarcely possible to come to any other conclusion than that the "lunatic" described above in the gospels was simply epileptic. The poet Lucretius describes one labouring under this malady in terms exactly similar:—

> "spumas agit, ingemit, et tremit artus

> Desipit, extentat nervos, torquetur."

Numerous examples answering to this description of "the lunatic," might be found in the epileptic wards of every asylum. We think it clear, therefore, that the Evangelist did not affix to the word *σεληνια-*

ζόμενος by any means the wide signification now attached to our corresponding word "lunatic." Nor did he, in applying to it the meaning of epileptic, use it in an arbitrary and unauthorized sense. Galen so uses it. And we find, on reference to the learned and most valuable lexicon of Peter Mintert, that this is the only meaning he attaches to it. His words are "σεληνιαζόμενοι, *lunatici sunt, qui epilepsiâ, seu morbo comitiali laborant.*"*

We have shown before that in *one* sense St. Matthew does not distinguish demoniacs from lunatics at all; and we think the above remarks prove that when he does represent them as he does in chapter four, verse twenty-four, as two distinct classes, the difference between them amounts to nothing more than the difference between our maniacs who do *not* suffer from epilepsy, and those who do.

We think, then, that it is proved beyond a doubt, that unless demoniacal possessions and insanity be considered analogous, no single instance of the cure of what we should now call an insane person is recorded in any of the gospels. Not even in the most general manner is any such cure alluded to. And this not only paves the way for a further investigation into the meaning of the disputed word, but also, as we hope to show, aids very materially in determining what that meaning is. For if, as has been often asserted, and asserted too in terms of no measured contempt for the intellect or the principles of those who hold the contrary, the demoniac laboured under an affliction totally different from any mere mal-organization or lesion of the brain, how, then, is the proved omission in that case of any miraculous cure of insanity from all the gospel histories to be explained? Such an omission can only be accounted for on the supposition, either that no insane persons existed at the time; or, that though they did exist, none were brought for cure; or, that they were brought, but our Lord did not heal them; or, that he healed them, but the fact was not deemed important enough for special record or for general reference.

A moment's consideration of the subject will suffice to show that any one of these suppositions is too unreasonable to be entertained.

It is impossible to believe that the Jews alone, of all the nations on earth, were exempt from this fearful and mysterious malady. In a condition of society constituted as theirs then was, there was every

* That the word σεληνιαζόμενος, or lunatic, should be limited in its application to epileptics only, and not extended to all the insane, is not to be wondered at. It was generally believed by the ancients, and the belief still lingers in some quarters, that the moon has a decided and especial influence upon epilepsy: and that belief seems to be incorporated in the word σεληνιαζόμενος.

Dr. Guislain says that though "mania recurs by periodical returns, there is no *regularity* in these manifestations. It is in cases of epilepsy that the greatest regularity is observed."

It was therefore natural that a word which expresses that belief in lunar influence should be applied, as we see it was applied, to epileptics.

influence at work upon them calculated to excite and produce insanity.

The glory of their nation had departed. They were crushed beneath the yoke of the Roman power—a power regarded by them with mingled feelings of fear and hatred, of horror and aversion. Universal attention was eagerly strained to catch some signs of coming deliverance—signs of the advent of that period predicted by their prophets, and believed to be at hand, in which, as they hoped, they were to rise again to more than their ancient greatness, and their enemies and conquerors were to be for ever broken and destroyed, "beat small as the dust" beneath their feet, and swept away before the whirlwind of their power. Yet no signs of such deliverance could be seen. Proofs of their degradation and of a miserable thraldom met them in all directions.

The Roman tax-gatherer came with his hated exactions, and if they dared to resist him, the majesty of the Roman law was avenged by the magistrate or the soldier. Such visions as these were the things that met their gaze, whilst they strained their eyes looking for signals of returning *glory*. It would almost seem to them as if the words of their prophets had been spoken in vain. Their elated hopes of dominion would appear but as the dreams of delirium. When they looked for deliverance, some Roman pageant, like a mocking fiend, laughed at their misery, and poured scorn upon them. When they conspired and struggled to be free, their conspiracies were detected and crushed, and they sunk only to a deeper slavery. They fell down exhausted by these convulsive efforts; their chains were fastened more firmly around them. It is impossible but that multitudes should be driven to madness by this degradation and misery. But in addition to this, the deepest corruption spread and festered in the very heart of the people. There was unbridled licence of lust amongst them; hatred one of another, divisions, jealousies, cruelty, sensuality. Could the national conscience sleep amidst all this? It is impossible. The remembrance of what they had been, when God's laws had been more regarded amongst them; the knowledge of what they were then, when all law, human and divine, was hated and trampled on, could not fail, however their moral sense might be deadened, to rouse them to remorse, and to haunt them with all ghastly apparitions and fears for the future. Palpable and monstrous distortions of mind *must* have arisen out of such a state as theirs. Having lost their faith in God, they had come to believe in lying vanities. They bent their attention to forbidden science to see, it may be, whether they could not gather hope from that. They believed in, and consulted demons. It is from facts like these that Dr. Lightfoot accounts for the demoniacs that there were amongst them. But these are the things which always overthrow the balance of the mind, and lay it open to the incursions of disease.

These are the causes which invariably drive men to madness, and sink them and their offspring to a helpless idiocy, and to incurable mania. The supposition, then, that there were no insane persons existing in the time when our Lord appeared in Judea is most unreasonable.

And it is equally unreasonable to suppose that there were instances of mania amongst them, but none were brought to Christ for cure. They brought their sick, the poor paralytic, the lame, the blind, the deaf, the dumb; they even besought Him to come to the chamber of death, and did not doubt that if He spake the word, disease should be arrested, and the dead should live. Is it possible, then, to conceive that they would leave their maniacs, the victims of a malady the most painful, the most terrible that man can be subject to, the most dangerous also to society, and not bring them also and lay them at the feet of Jesus, beseeching Him to heal them? We conclude, therefore, that they did bring to Him the insane; and the supposition that they were brought to Him in vain, is too irreverent to be entertained. He healed disease; He opened blind eyes, and unstopped deaf ears; He loosed the tongue of the dumb; the very grave gave back its dead at the bidding of His word. Who, then, dare doubt His power to dispel the delusions of a diseased understanding, and to restore sanity? or who, that has read His life, dare doubt His willingness to exercise that power? It is impossible to resist the conclusion that He healed the insane, as many as were brought to Him. And this work would be so wonderful, so unheard-of a miracle, so mighty a proof that He was indeed the Restorer of mankind, the Divine Logos, the Light "which lighteneth every man that cometh into the world," that it is impossible that the Evangelists, who delight to set forth His greatness, should all of them have omitted to record a miracle like this. But, unless the word demoniac have this meaning of insane, they *have* omitted it; there is not even an allusion to it. This fact gives to our mind no small degree of probability to the opinion, that the two words mean the same thing;—a probability which is further heightened by this: that all we read of the character and acts of these demoniacs corresponds in a most marked degree with all we know and witness of the character and acts of the insane.

In adducing our proof of this position, we shall confine ourselves entirely to the meaning, and as nearly as possible to the very words, of the Evangelists' descriptions of persons possessed.

They were "fierce," ungovernable, violent, possessed of unnatural strength. They were "bound with fetters and chains," which "they plucked asunder, and broke in pieces." "No man could tame them." They dwelt naked (for "they tore their clothes" from them) upon the mountains and "among the tombs," and "no man durst pass by the way." One is spoken of as crying (κράζων), uttering screams, and tearing himself with stones. We add nothing to this description except to observe, that the language which they are represented as addressing to our Lord is characterised by the same irrational wildness as their acts. "They ran to Jesus, and *cried with a loud voice*, and said, What have I to do with thee, Jesus, thou Son of the Most High God? I adjure thee by God, that thou torment me not." We venture to say that if this description, selected in the above words from one or other of the gospels, were to be met with in any other book, any number of persons who should read it, or hear it read, would, without hesitation, unanimously pronounce the men whom it represents to us to be maniacs. Be it remembered that there were no institutions then for the mitigation of their disorder. They were probably treated with a mixture of fear and violence, which would naturally increase their fury. It was natural, therefore, that they should fly to uninhabited places, and that men should not dare to go near them. The case would be the same with our insane, if the treatment were the same. It has been so, within every man's memory, before that modern and milder treatment came into operation; that system of moral instead of mechanical restraint, which is one of the wonders and glories of an enlightened medical science. Even under this milder and wiser system of ruling them, their character and language present many points of strong similarity with the above descriptions. They often burst out into acts of the wildest violence; they often believe and fear that those who approach them as friends are come "to torment them."

We are bound, however, to admit, notwithstanding the great, we might almost say *perfect*, similarity there is between the demoniacs and the insane, there are still very considerable difficulties in the way of that interpretation which would represent them as one and the same. These difficulties, which we hope to remove in the course of this discussion, arise from some peculiarities in the Evangelists' language, not easy to reconcile with modern scientific theories of insanity. The demons, that are declared to "possess them," are described as uttering adjurations to our Lord, and exerting violent acts of power over their victims. Our Lord in return is spoken of as addressing His words to them, giving His command to them to depart, or His permission to enter a herd of swine. Now even allowing that you are not to expect the same kind of description from the Evangelists that would be presented to you in a medical treatise—admitting that their purpose was entirely different—still there is a something in the language which speaks of men as "possessed by devils," which seems utterly at variance with all our ideas of mere insanity. This difficulty is sometimes attempted to be got rid of in a very summary manner, by the answer that our Lord and His followers described the malady in such terms out of regard to the prejudices of the Jews. We cannot think that this is an adequate or even a fair reply to the difficulty. For it implies that Christ and His disciples deliberately countenanced an opinion which they knew to be false; that they fostered superstition though their professed object was to proclaim only truth.

We may be quite sure that they would never sanction any falsehood—they would never countenance any superstition. We may be quite sure, also, that when they spoke of the miserable victims of madness, as *δαιμονιζόμενοι*, they had some real and deep meaning in it. And we believe that only by taking this view, and meeting the difficulty which the words present fairly and honestly, shall we be able to find any answer to it. No argument was ever yet really strengthened, but rather weakened, by disingenuousness and evasions.

There lay a very awful truth under their words—a truth which, in our opinion, is scarcely held in view at all, but to a great extent obscured and overlooked, in the meaning so often attached to their words, of a real, visible, palpable demon being within the men. What the Evangelists believed, and meant their readers to believe, was this, that all evil, especially such evil as affected the spirits and minds of men, was the work of the devil; and they represent Christ as the Deliverer from this evil. Now if they had represented Him as healing men's bodies only, whilst He passed over the diseases of the mind, they would scarcely have given a full idea of what the Deliverer was — the Destroyer of the devil's power, the Restorer of man's true humanity. To attribute then, as they do, the malady of insane men to the "possession" and power of Satan, is only speaking in accordance with the view given above—in accordance with the whole spirit and teaching of the Bible—in accordance also with their own expressed teaching with reference to the diseases of the *body*. For, in recording the case of that poor "daughter of Abraham" bound, and bowed together with the spirit of infirmity eighteen years, it is expressly stated, that "she was bound by Satan." He is described in another place as possessing "the power of death," *τὸ κράτος τοῦ θανάτου*. To this it will naturally be answered that though disease and death are represented as the work of the devil, yet we never read of any diseased man as being "*possessed* by the devil." Of course we never do. The language would be absurd and false in such a case. For disease affects only the *body*, the outer shell, and not the real ego of the man. But insanity possesses the mind, the actual personality of the man. Therefore in the one case it is simply said, Satan *hath bound* the body; but in the other, the man is possessed with him. And it would be just as absurd to infer from this language, in the one case, that actual demons dwelt within the man, as it would in the other that diseased men were stricken or bound, or the dead cut off, in every case by an actual stroke or personal act of the Evil One. What is really meant is this—that disease and death are the effect of that sin which the devil tempted man to commit, and still tempts him to perpetuate; and that insanity also is the result of the same malignant influence, exerted on the mind as well as the body. This, indeed, is all that can be drawn from the Scripture words on the subject. We never read of one single case possessed by *Satan*, the actual, the real διάβολος, but by δαίμονες, or δαιμόνια — evil *influences* proceeding from the Prince of Evil. There is certainly nothing in the word δαίμων itself to indicate more than this —nothing to indicate, necessarily, a personal existence. It is used in one case, at least, by the Septuagint translators in no stronger meaning than this, rather indeed a weaker, as the equivalent of the Hebrew אֱלִיל *res nihili*, any vain, perishable thing, particularly idols.

And everything which the Evangelists predicate of these demons is perfectly easy of explanation on the supposition that the δαίμονες were not personal existences. It is true they are spoken of as casting their victims into the fire, &c. But every language has forms of expression analogous to this. It is just what we should expect in an Eastern language; that the words and deeds of a man acting under the impulse of insanity should be attributed, not to the man, but to the influence by which he was impelled. Free as our own language is from all tendency of the kind, we have analogies even in *it*. We personify anger; and men's words uttered in passion are overlooked and forgiven, as being not his own words, but those of that furor that possessed him. We say of the follies and inanities uttered under the influence of wine, "It is the wine speaking." The language of the Evangelists means no more than this—a man utters wild words, or does deeds of folly and madness, but the man's mind is not under his own control; an evil influence possesses him, therefore his deeds and words are not attributed to himself, but the evil power, or δαιμων, that has rule over him. It is just the very language we should expect to find in the Gospel descriptions of the men; the very language that would appear most natural and proper, more especially when we take into consideration the fact that insane persons often speak and act under an impulse against which their real self rebels, but is too weak to resist. We quote as an authority for this assertion the opinion of Dr. Guislain on the subject, from the analysis of his work which appeared in former numbers of this journal:—"I have known patients who have said to me, 'Something, I know not what, an electric force, perhaps, *compels* me . . . . . I *must* act in opposition to my intentions. Others say, 'There is in me some one who is not myself—who drives me, and forces me to act.'"

Every one at all conversant with the insane must have seen cases of this kind; men possessed of a sort of double consciousness; the actual ego of the men feeling itself fettered, or driven onward by some strange impulse, and perfectly conscious of the thraldom, yet not having strength of will to resist it.

The tenour of these observations is to prove that the language employed by the Evangelists in speaking of the demoniacs, though at first sight it presents us with some difficulties, is not, on a fuller consideration, *inconsistent* with the theory that the insane and the demoniacs are one. There is nothing in it to overthrow that theory; so that the arguments which we employ to show the analogy between the character and acts of the insane and of demoniacs remain in their full force, unassailed and unassailable.

We shall proceed now to offer some evidence, that in giving to the word the signification of maniac or insane, we are not imposing upon it an arbitrary meaning of our own. We are not without proof that this is the sense which the Jews and the writers of the Gospels themselves put upon it. We have seen in a former part of this Essay, that the Evangelist St. Matthew employs this, or an equivalent term, as synonymous with the word Lunatic, or Epileptic. Now, if an epileptic person were demoniac, the inference is clear that the writers of the New Testament did not employ the word to signify a supernatural disease. And since their application of it shows that it has also a wider meaning, and that it refers to a class of diseased persons of whom the epileptics are but a small portion, how irresistible the conclusion that that class to whom they applied it were simply the insane. This conclusion is confirmed by the fact that, in St. Matthew's account of the healing of one of these demoniacs, he speaks of the man after his restoration to health, as "sitting at the feet of Jesus, clothed, and in his *right mind:*" σωφρονοῦντα is the word he employs on this occasion — a word which clearly intimates that, according to this writer, his state of mind was unsound before, but the miracle of healing had given back its sanity again.

The people also affixed this meaning to the term. On more than one occasion we read of them uttering these blasphemous words to our Lord,—"Thou hast a devil:" an expression exactly analogous to the words, "Thou art demoniac." Now they apply this expression to Him, not because of any supposed wickedness in Him, but because His language appeared to them unintelligible, incoherent raving. He said, they went about to kill him. They thought this the dream of a madman. He told them, by implication, that they were not Abraham's children. They thought it the language of folly and delusion, and again apply the term to Him. He told them, if a man kept His saying, He should not see death. This seemed to them madder than all.

Abraham was dead, and the prophets. It seemed to them the wildest folly; and they replied, "Now *we know* thou hast a devil." On the fourth occasion they clearly explain their meaning,—"He hath a devil *and is mad*," καὶ μαίνει,—He was under delusion, a wild and mad enthusiast, deceived, and a deceiver.

This καὶ μαίνει of theirs is the very term applied by Festus to St. Paul, when he believed him, as *they* believed Christ, to be speaking the words of a wild delusion.

We see, therefore, that the expression, "Thou art demoniac," is only a Jewish way of declaring that a man is mad, deluded, wild, insane.

Before we conclude, we must say just one word in reply to a large class of persons, whose scruples we wish to respect, who believe that our view on this subject goes far to deprive our Saviour's most wonderful works of all that made them miraculous. We scarcely flatter ourselves that any argument of ours will carry conviction to their mind. We do not know what views *they* entertain of insanity. It may seem to them a very slight, unnoticeable miracle to heal it with a single word, as our Lord did. But for ourselves we must confess that to restore the demented to vigour of thought, to banish all mental delusions, to heal raving maniacs and hopeless idiots in an instant and with one single word of power, seems to us one of the grandest, noblest, most Godlike miracles we can possibly conceive of. It is a miracle which, to our thinking, has a much deeper meaning for *us*, and all generations of men—a meaning that appeals much more to our sympathies than that which represents Christ as ejecting from men actual, palpable devils. For in the one case, wonderful though the work is, He is but healing a disease which never, that we know of, appeared before, and never may appear again. Whilst in the other, He cures a malady which all men concur in believing to be the most fearful that can afflict humanity; a malady that has existed in every age of the world; a malady that may befall any one of us, since, to use the language of an eloquent and learned writer on insanity, "neither the genius of a Southey or a Tasso, nor the wit and vigour of a Swift, nor the tenderness of a Cowley, nor the piety and talent of a Cruden or Hall, can exempt men from its influence." In healing this disease, Christ appears as the Lord of mind and matter, the Restorer of our true and real humanity. He declares to us, by this miracle, that as it is the work of the Evil One to overthrow the balance of the mind and cover it with darkness; so it is *His* work to restore and give light. And all they whose lives and talents are devoted to the study of this painful disease, that they may mitigate the evils of it and remove them, have thus, in their noble work of mercy, the encouragement of His Divine example.

